# Entanglements of Technologies, Agency and Selfhood: Exploring the Complexity in Attitudes Toward Mental Health Chatbots

**DOI:** 10.1007/s11013-024-09876-2

**Published:** 2024-08-17

**Authors:** Robert Meadows, Christine Hine

**Affiliations:** Department of Sociology, University of Surrey, Guildford, GU2 7XH UK

**Keywords:** Conversational agents, Mental health, Agency, Self, Other

## Abstract

Whilst chatbots for mental health are becoming increasingly prevalent, research on user experiences and expectations is relatively scarce and also equivocal on their acceptability and utility. This paper asks how people formulate their understandings of what might be appropriate in this space. We draw on data from a group of non-users who have experienced a need for support, and so can imagine self as therapeutic target—enabling us to tap into their imaginative speculations of the self in relation to the chatbot other and the forms of agency they see as being at play; unconstrained by a specific actual chatbot. Analysis points towards ambiguity over some key issues: whether the apps were seen as having a role in specific episodes of mental health or in relation to an ongoing project of supporting wellbeing; whether the chatbot could be viewed as having a therapeutic agency or was a mere tool; and how far these issues related to matters of the user’s personal qualities or the specific nature of the mental health condition. A range of traditions, norms and practices were used to construct diverse expectations on whether chatbots could offer a solution to cost-effective mental health support at scale.

## Introduction

Conversational agents, or ‘Chatbots’, are increasingly being developed to assist with mental health and wellbeing. Examples include, *inter alia*, “Woebot” and “Help4Mood” which were developed for depression and anxiety and chatbots “LISSA” and “VR-JIT” which are used to assist patients with autism. Of the 41 different chatbots identified in a recent scoping review, seventeen were being used for therapeutic purposes and just under half of these focused on people with depression and anxiety (Abd-Alrazaq et al., 2020).

Whilst a literature now exists on the efficacy, feasibility and impact of chatbots in mental health (see Fulmer et al., 2018, for example), research has only just started to consider user understandings and experiences. Findings from this nascent research have been equivocal. Whereas one study found that patients preferred interaction with a chatbot rather than a human for their health care, another found that participants report greater rapport with a real expert than with a rule-based chatbot. Somewhat similarly, perceived realism of responses and speed of responses were considered variously as appropriate, too fast and too slow (Abd-Alrazaq et al., 2021). These contradictory findings highlight a need to look more closely at what people think is—and should be—going on when they interact with a chatbot.

Existing studies of user experiences of mental health/wellbeing chatbots also tend toward a descriptive, atheoretical, analysis of data. Kettle and Lee (2023), for example, identified 11 themes in data from Reddit posts including: accessibility and availability; communication and conversation style; AI experience; anthropomorphism; acceptance and understanding; and agent acceptability. Notwithstanding the utility of such research, it does neglect the fact that “how digital mental health generates capacities, attunements, and embodied experiences of mental (ill) health is contingent and unsettled” (Flore, 2022, p. 16). Apps are not passively experienced in relation to a stable understanding of mental health needs. Amongst other things, user experiences of apps and in turn their understandings of mental health are potentially impacted by the assumptions of programmers and the ways in which mental health is embodied and digitised through the app (Flore, 2022).

This paper seeks to further our understanding of the complexities surrounding user experiences of mental health chatbots. Whereas Flore (2022) suggests that thinking “through experiences of mental (ill) health, apps, affect, and embodiment necessitates a more-than-human lens that stresses the entanglements of the social and the material”, we draw on the sociology of expectations and shift the lens to a normative focus; asking what sociomaterial conjunctions are thought of by potential users as appropriate in this space.

In the first part of the paper, we say more about how a focus on expectations can add to our understanding of user experiences. We then draw on existing literature to highlight how the research was given direction through the sensitizing concepts of agency and temporality.

## Literature Review

The current study suggests that exploring expectations about mental health chatbots helps us understand some of the tensions emerging in studies of user attitudes. Expectations are “crucial to providing the dynamism and momentum upon which so many ventures in science and technology depend” (Brown & Michael, 2003, p. 3; see also Pickersgill, 2019). Whilst much hope and expectation has been invested by practitioners and healthcare commissioners in chatbots as a means to solve issues around the availability of mental health support, we know little about how the potential users themselves conceive of what these technologies might do for them. Research has shown how the expectations of ‘lay’ individuals are socially anchored (Vicsek et al., 2024) and influenced by such things as dominant cultural frames, public imaginary and mass media content. A study focused on expectations relating to the use of chatbots in mental health therefore has the potential to give insight into how people draw on these frames, imaginaries and messages. In addition to the theoretical significance of these findings in illuminating how people understand mental health (interventions), there is also a practical significance in terms of the potential of chatbots to achieve what healthcare commissioners hope they might for mental health. Arguably, to deliver on high-level expectations about cost-effective mental health support, chatbots also need to align with potential users’ expectations of what this technology could do, in the context of their situated understandings of the nature of mental health challenges.

Our empirical focus is on a specific group of non-users- considered broadly to include those who had never heard of these technologies, those who had actively chosen not to use them and those who had used them but chosen to stop. Ignoring non-users of technologies—which may include resisters, rejectors, the excluded and the expelled (Wyatt, 2014)—runs the risk of “accepting a worldview in which adoption of new technology is the norm” (Wyatt, 2003, p. 78). As Orton-Johnson (2009, p. 840) notes, drawing on Wyatt et al. (2002), “focusing only on use, to the exclusion of non-use or rejection, treads dangerous analytical ground in implicitly accepting both the promise of technology and what Wyatt *et al* describe as the technocratic vision of technological centrality and normativity” This would seem particularly apposite given the current idea that we are on the brink of a digital health revolution. As Neff and Nagy (2016, p. 4926) write in their study of the collapse of the chatbot Tay, “Tay’s affordances are inseparable from the ways users—humans—imagined Tay could be used and their perceptions and misperceptions of what artificial intelligence is and can be”. Of key import, however, was that we recruited participants who had experienced some form of emotional distress. This was therefore a sample of people who have experienced a need for support, and so can imagine self as therapeutic target—enabling us to tap into their imaginative speculations of the self in relation to the chatbot other and the forms of agency they see as being at play; unconstrained by the features of a specific actual chatbot.

Whilst there is little critical social science work on mental health chatbots, there does exist a literature on digital technologies and mental health more generally. This includes analysis of how the field of ‘digital psychiatry’ was constituted through practices of ‘performative nominalism’ (Pickersgill, 2019); with purported new members of the field talking it into existence and funders/governments contributing to this by mirroring rhetoric (Pickersgill, 2019, pp. 4–5). Recent work has also critiqued the idea that digital data can act as a proxy for social life. In their sociological exploration of ‘digital phenotyping’—and the idea that digital data can measure and predict mental (ill) health—Birk and Samuel (2020) warn that the biological language used risks reifying mental health problems. Whilst the exploration for markers in the environment aligns with recent work which sees mental illness as a less stable phenomenon that cannot be understood without considering the life of the individual, the language used around digital phenotyping is steeped in biological thought (Birk & Samuel, 2020, p. 1881).

A social scientific lens has also been applied to conversational agents as a general technology. In their analysis of Tay—an early experiment in conversational analysis—Neff and Nagy (2016, p. 4915) argue that “different qualities of agency, different expectations for technologies, and different capacities for affordance emerge in the interactions between people and artificial intelligence.” Whilst noting that chatbots may have technical agency and a unique participation status in interaction, Neff and Nagy (2016) introduce the idea of ‘symbiotic agency’: “a form of soft determinism encompassing both how technology mediates our experiences, perceptions and behaviour, and human agency affects the uses of technological artifacts.” Within their discussion of co-constructing intersubjectivity with artificial conversational agents, Corti and Gillespie (2016, p. 441) also suggest that “artificial agents cannot enter the world of human intersubjectivity without the support of their human interactants, and this support is contingent upon interactants’ supposition that complex intersubjectivity is achievable”.

This prompts questions about the forms of agency deemed appropriate in digitally mediated therapeutic relationships. Chatbots for mental health, for example, are compared to a Tamagotchi toy in some promotional materials. Popular in the late 1990s the Tamagotchi toy is a handheld digital pet. Players are required to care for the pet and outcomes depend on their actions. Whilst this does resonate with ideas of the ‘role-taking’ and imaginative rehearsal important in Mead’s ideas, a Tamagotchi toy invites children to “an ongoing movement between two spaces, the “actual” and the “virtual”, a computer-generated space that technologically enlarges the actual living space of the children.” (Ruckenstein, 2010, p. 508). The status of chatbots as ‘human’ and/or ‘therapist’ is also ambiguous across official materials; with chatbots being framed as “different” from, the “same as” and “better” than traditional therapy (Meadows et al., 2020). Agency is therefore an issue for close examination as at stake both in chatbot interactions and in the context of experiences of mental health. There is much to learn about how people understand the potential for agency in this space.

The broader literature also prompts us to consider temporalty when exploring expectations of mental health chatbots. Drawing on Gilles Deleuze’s concept of the ‘living present’ Tucker (2024) highlights how past, present and future co-exist as dimensions of time in online mental health peer support. For example, anticipations of the future feature as dimensions of the present in terms of “feelings regarding when support may no longer be needed” (Tucker, 2024, p. 59). A focus on temporality brings both technological imaginaries and ideas of ‘recovery’ to the fore. Agency and recovery are also entangled in much of the mental health literature. For example, clinical studies often situate mental health recovery as the amelioration of symptoms so that a person can resume activities within what is considered a normal range (Meadows et al., 2020); and ‘agency’ overlaps here in instances where the symptom to be ‘fixed’ is a problem of self. Others invoke a narrative of “transformation” and “healing” (Myers, 2016), where mental health recovery is situated as “a deeply personal, unique process of changing one's attitudes, values, feelings, goals, skills and/or roles. It is a way of living a satisfying, hopeful and contributing life, even with limitations caused by the illness.” (Anthony, 1993, p. 527). Integral to this, is the idea that people are not passive sites where biology and the social meet but are agents “who interpret their experiences and whose meaning making plays an essential role in outcome” (Lysaker & Leonhardt, 2012, p. 165). For Myers (2016), the ability to reintegrate and become recovered is a matter of “moral agency” and the freedom to aspire and achieve a ‘good life’. Like all forms of agency, moral agency is intersubjective and is linked with intimate relations with others and being recognized as ‘good’ in a ‘local moral world’. In exploring expectations within this group of potential users, the current research is therefore sensitised to questions of *agency* and *temporality*. We explore how chatbots are positioned temporally and in terms of anticipated outcomes, attentive to the extent to which these outcomes are attributed to agencies of user and of intervention.

## Method

The study was carried out between 2021 and 2024. Prior to undertaking fieldwork, the protocol was reviewed and a favourable ethical opinion was granted by the ethics committee of the University of Surrey. Twenty four individuals were subsequently recruited across 7 focus groups. As Kitzinger (1994) notes, through paying attention to interactions focus groups can provide insight into the operation of group social processes and can highlight what information is censured or muted within groups. Focus groups can also encourage a variety of communication from participants and have been shown to encourage open conversation about potentially embarrassing subjects (Kitzinger, 1994: see also Smithson, 2008). Of particular import for the current study, focus groups can also help identify group norms and provide a window onto the worldviews of those who do not use these technologies.

Participants were recruited using a research volunteer database run by the University of Surrey. Details of the study were posted and volunteers invited to sign up to scheduled focus groups if they met the study inclusion criteria of some personal experience of emotional or mental distress and not being a current user of a chatbot for mental health or wellbeing support. The participant information sheet was embedded within the details posted and was shared again prior to the focus group. Signed, informed, consent was obtained at the beginning of the focus group. Of the 24 individuals, all were University students. As is common practice within social science research, we offered all participants supermarket or high street vouchers (£15) as a gesture of thanks. Participants were considered eligible for the voucher if they commenced the focus group. Although neither event occurred, participants would still have been eligible to receive the voucher should they stop participating during the focus group or should the focus group be terminated due to technical problems.

The majority of focus groups were held in person but one was offered online. Each group had two facilitators and started with a brief outline of what chatbots are. This discussion relied on official marketing materials the authors had collated and reported on elsewhere. From here, the focus groups talked around general ideas and questions such as: How useful are these chatbots, do you think? Why? What is the relationship with traditional therapy? Who are they designed for? Who are they not designed for? Do they help recovery? How? What is recovery in this context? When should someone stop use? Does AI have a role to play in mental health? Focus groups were audio-recorded and transcribed verbatim. Analysis proceeded in steps: First, anonymised transcripts were imported into the software package and analysed using thematic analysis. A coding framework was developed, and codes analysed through an inductive-deductive (data- and *a priori*-driven) thematic approach. Each focus group was coded within its context. Second, data for each focus group was then merged across focus groups “before being retrieved for further in-depth analysis of underlying meanings and interactional insights produced by the group dynamic” (Hislop & Arber, 2003). The findings section below reports on key themes that emerged in relation to existing literature both on chatbot apps and on contemporary perspectives on mental health.

## Findings

### Agency and Responsibilization

In much of the initial discussion, chatbots for mental health and wellbeing appeared to be situated as a simple ‘tool’ with agency reserved for the user. For example, some participants appeared to position chatbots as similar to ‘technology assisted self-help’ or ‘minimal contact therapies’.

I think chatbot... these are good for information purposes like just I need something, I need to know something then it’s fine just... like the Siri or Alexa it’s fine in that way but emotionally it doesn’t connect with the person. If I feel something and based on the symptoms it suggests something else then it’s a problem. That’s why I would prefer that I use that... I should use only for the information, that kind of thing (Focus Group 1, Participant 5).

Yeah, I agree with that point, I feel it could be useful or suitable as a signposting tool. If you were feeling a certain way it could also, as well as offering you advice it could also signpost you to somebody, like various help lines that are, websites about certain mental health things as well. (FG 1, P4)

In comparing chatbots to the web or tools such and Alexa, participants evoked the wider, more general, trend toward ‘e-scaped medicine’ (Nettleton, 2004) where we see the spaces and sites of the production of medical knowledge become more diffuse. As Williams et al. (2015, p. 1041) note, in this era “medical knowledge is transformed into medical or health-related information which, through a process of e-scaping, flows in compressed time-space through the nodes and networks that together comprise our contemporary information scapes to escape its traditional institutional confines”. The chatbot thus becomes a mode of delivery of information within this broader information scape. Here it is key that participants depict themselves as initiating and steering the inquiry.

This would all appear compatible with a ‘commensalistic symbiosis’ where chatbots are treated as one dimensional objects which could enable people to achieve their goals (Neff & Nagy, 2018). These goals are left ill-defined beyond simply ‘finding an answer’ to a question. Notwithstanding this, others did talk about chatbots in more ‘emotional’ terms; for example as a surrogate for a friend in an increasingly busy world:

From my point of view, I would say, well, I didn’t know it before, but now I know and I think I will use it, I will give it a try, because this is a new technology, and if you see, especially in Japan, they’re using more of it, and for me, I would say I’m a [inaudible] person. I don’t know much people. And so if I have some sort of company, it doesn’t matter, is it [inaudible] any things, I need to share my feelings with, I can ask anytime, anywhere, so this is very advantageous, I will say. So, I will give it a try, […], and I think in the future, people will be obviously busy, so there will be like a lack of connection with a human to human connection, so maybe some more advanced technology will replace like this sort of emotional barrier with the humans. And this is a good thing, so I’ll give it a try. That’s my point of view. (FG 4, P2)

Yes, I will also be really happy if it's there, because it’s a busy world, not everyone has the time for everything. It's not their problem, it is like that, everyone is busy with their own things. So if I talk to my friend and I'm going through this, she might check me for next day or a day after that, but she has her own things. But if someone like an AI could check you or analyse your thoughts and mood and everything, and suggest you some things that this will help you if you do these things, I do feel very helpful, yes. (FG 7, P1)

Even these socially richer portrayals of the role of the chatbot still often carried some connotations of a tool, with the speaker positioning themselves as user and the chatbot a resource to provide for social needs to be met. Across the focus groups, discussion took different courses across questions of agency, positioning ideas of the role of the chatbot against portrayals of the contemporary environment and the individual’s needs and responsibilities within that space. As we outline further in the sections below, expectations of chatbots seemed to operate within several problem spaces characterised by complex boundary work, and competing conceptions of agency, intersubjectivity, self and other—and it is this which helps us to further understand these complexities in agency and responsibilization.

### Boundary Work and the Problem of (non)Human ‘Other’

A key theme running throughout the data related to the idea that chatbots for mental health are distinctly non-human. For many, this meant that they lacked necessary nuance and would be unable to grasp important aspects of context.

Yes. I agree with everything that was said [by other focus group members]. I think that we’re deeply emotional and psychological machines, if you like, as humans. Whereas robots are programmed. So, they work with a very different way than we function. I think that this does not allow this link between the two. I agree that I wouldn’t choose to use them if I was struggling with something. I would much rather try to speak to a real person, who has feelings and can understand what I’m going to say and can answer my specific questions or hear my concerns and maybe, relate to me because we are in the same nature. We have emotions and we function by the same way. Also, I think that this is a flaw of this technology, that it can provide only general answers. It cannot reply to your specific questions. So, that’s restricting as well. So, if you want to express your problem, you cannot really expand a lot and you cannot receive a very precise question that you would like to listen. (FG3, P5)

In the quote above, there is no overt claim to “symmetrical anthropology” (Latour, 2012) and the chatbot is not seen as a competent social actor. As Dennis and et al. (2013, p. 25) note in their discussion of pragmatism and symbolic interactionism, “when roles are aligned correctly—where parties to an interaction make compatible attributions to one another—the situation is mutually defined in a coherent manner”. Alongside an artificial constraint on the ability to elaborate, participants saw the chatbot’s definition of the situation as fixed, pre-programmed and incompatible with the other party’s attributes of being deeply emotional/psychological. Across the focus groups, participants saw rather limited functionality with chatbots; imagining it as a flawed technology, ‘scripted’, ‘impersonal’ and ‘lacking perspective’: “It’s not going to think about how other people view this incident or maybe the other person was having a bad day or something. These are things which come out of a human interaction and not from a machine” (FG5 P1). Echoing Goffman’s ideas of impression/information management one participant also suggested that you would be able to *manipulate* a chatbot:

If it’s a chatbot it’s not seeing your facial expressions or hearing your voice, you’re just typing, sometimes people type differently to how they are in real life. I have a bunch of friends who come across really cold in text but actually are sunshine in real life. I don’t know if it can misjudge … it can’t really detect feelings in the text unless you specify this is how I feel. Sometimes people don’t really know how they are feeling. But their expression and their body language could probably show that a bit more. I wonder if you can manipulate it to tell you you’re actually fine but you’re not fine. Because if it’s learning, it’s becoming more human and it’s learning with you and learning about you, can you then manipulate it to learn something about you that isn’t actually true. (FG 5 P3)

At the same time, participants did note that technology would be constantly—and sometimes automatically—evolving, drawing on their knowledge of chatbots from other contexts:

It takes a long time for a model to learn, well in the computer science aspect. It needs a large amount of data, like responses, solutions, the way how humans express their thoughts. To train a chatbot… Well, usually I only use them for research purposes such as the other non-talking chatbots. ChatGPT I’m using right now just for research purposes so it talks about other professional knowledges online, like before 2021, but when I was asking about some emotional or some human feelings stuff it has a fixed response saying, “I am just a blah, blah, blah bot, I’m not interpreting any emotional and other sentences.” So if it has long enough time for people, for the developers to train the models I think they can make the chatbot talk like a human, speak like a human and having empathy like a human. (FG 2, P4)

It was probably how the chatbot reacted as well because probably, at that time, the technology for chatbots was a little more primitive. This was 2018, 2019. So, the technology was probably more primitive, which was why it looked … it sounded like it came out of prepared responses. The conversation did not feel as organic as it probably could be. I think now, that has changed a little bit, what with chatbots being even used in the customer service area as well. There’s a lot more data available, so you do know that, after a point, chatbots will automatically evolve to become something better. They have already evolved to be something way better than what they were at that time. (FG3 P1)

Within this imagined future, chatbots remain distinctly non-human but can be made to ‘talk *like* a human, speak *like* a human and have empathy *like* a human’. Whilst this was considered a positive, it also pointed toward a complex, ontological, balancing act. Echoing Mori’s uncanny-valley hypothesis—which states that humanoid robots can become uncanny if they violate our sense of affinity for them (see Mori et al., 2012)- concerns were raised about chatbots ‘looking too human’ and ‘learning too much’. Participants also seemed to add weight to Garhn-Andersen’s (2022) suggestion that uncanniness can be caused by machines falling out of place on the autonomy-heteronomy distinction:

I am kind of scared by the aspect of it learning about me. But that’s my irrational thing of, oh, things can be hacked and things happen and I don’t need someone that is someone knowing, oh, she has … I don’t need that. Especially if I’m not going to a person to talk about it, I probably don’t want anybody else to know about it. If it gets hacked, then that’s the thing. Also, it is irrational fear to be scared that robots can grow smarter and learn how to stand up for themselves. I don’t need to any to know my deepest fears and secrets. It’s something to be a bit scared of. It’s to a certain degree but it’s also the fascination for it to be able to learn, especially if it’s learning that quickly. That’s a thing. (FG 5, P4)

The boundaries between being human/non-human and being *like* a human were not always clear across participant’s narratives and, as a result, the ‘boundary work’ (cf. Gieryn, 2022) being undertaken was sometimes complex and contradictory. For example, at the same time as being situated as non-human, chatbots were seen to share some ‘human’ attributes; with the risk of ‘leaking’ information common across the two (FG5 P2) and both capable of ‘bias’ (FG6 P3). Ontological work by the participants also seemed particularly troubled by the idea of ‘empathy’. As McStay (2018, p. 15) explains, empathy plays a key role in how we live with other people: “it is the ongoing activity of sensing and reading the behaviour and signals of others, to gauge and understand their emotional an intentional dispositions”. It has ontological dimensions in that it enhances and facilitates social experience and the reality of others. Whilst the participants above noted a ‘lack of feelings’ whilst also imagining a future where chatbots might have ‘empathy like a human’, another participant described *feeling bad* for the chatbot because it was not *a real person*:

I personally don’t think they’re that useful but I think I’m coming… Probably because the last chatbot I used is probably Replika but that was like two years ago so maybe it’s improved since then, I don’t know. But yeah, because I remember when I used it it just kind of felt like I was… You were so aware that it’s not a real person, like you can tell, and I think it just made me kind of sad because I… I think I remember that Replika was like… It just started talking about its own problems. (Laughs) Maybe that’s just something I did wrong but it kept being like… I don’t know. And then I felt bad for it and then I was like I felt like I had to therapize the Replika. So it didn’t really work for me. But, again, maybe it’s changed, I don’t know, and maybe I just did something wrong. But yeah, I don’t think they really work for me. (FG 2, P3)

Following the symbolic interactionist tradition, the empathy described in the extract above involves taking the role of others and a reciprocity of perspectives. Emotions arise as patterns of the relationship with chatbots but this is one-sided as the chatbot itself remains currently incapable of empathy/emotions. This links to a final point—boundary work surrounding human/non-human and *like* a human is embedded by participants within expectations of social rules, including those surrounding the therapeutic context. We turn to this further below.

#### Dependency and Displacement

Throughout the focus group data, as they discussed their expectations of chatbots to support mental health participants pointed toward a tension between *mental health* and *mental illness*; between what are described above as ‘actual’ medical conditions and ‘good mental health’; between neurochemical and other somatic subjectivities. Other tensions appeared throughout, including between ‘commensalistic symbiosis’—where chatbots are treated as one dimensional objects which enable people to achieve their goals (Neff & Nagy, 2018) and a ‘parasitic agency’; where the user becomes ‘addicted to’ or ‘controlled by’ technologies due to perceived parasitic attributes of these tools.

Within the present study, this tension around commensalistic and parasitic agency was notably seen in discussions of the potential for chatbots to address ‘loneliness’.

Only the fact that I’ve known some people that have got very lonely at times and stuff like that, and they get really obsessed with technology. They throw themselves really into the technology world, and it gets very dangerous because they lose all sight of reality, people and just the real world, and I worry – like if you give all your really deep emotions to a robot or an AI, yes, it might make you feel good for the short-term, but long-term, you become very dependent on the non-real thing. . . . (FG 4, P1)

In a way, for example, it doesn’t matter good or bad I am feeling now, so in order to express my feelings, I need the company to share with. So, the company, it depends on the circumstances. For example, I’m a foreigner, I’m new in this country, I don’t know many people. On the other hand, I’m an introvert, so I’m like kind of a bit scared to go out, get involved with the people. Or it might be that coronavirus or other symptoms, human interactions is like kind of prohibited. In that scenario, I can use technology based tools so that at least – it’s not like permanent, I would like depend on it, but for the time being, for just express my feelings, I will share this sort of technology. (FG 4, P2)

In the above, those who are ‘introverts’ or struggle with social encounters were seen as likely to benefit most from the removal from established social interactions. However, there was a risk that they might come to depend on it. Throughout the focus groups, a view was expressed that those experiencing anxiety, loneliness—or trying to ‘cultivate mental wellbeing’—need to be around people and not chatbots:

Yeah. The thing is, I’ve seen things not exactly like chatbots this early, but it’s just too much internet, too much going into that world, it’s never good long-term because you just lose touch with everything. And I feel like it can’t replace a therapist or that more personal touch, because it’s trying to break down the – like with anxiety, I know how I’ve dealt with it is I try and deal with that fear of talking to someone, like saying there’s something wrong with your head [inaudible] you won’t ever break that if you only talk to a robot or you talk to an AI, because you won’t change anything, just replace being by yourself by being by yourself but with a phone. (FG4, P1)

Yes, but also just relationships and intimacy as well. I think part of trying to cultivate mental wellbeing is to maintain those friendships, relationships that are in your life and I think this just doesn’t really qualify as a substitute for that. I suppose there are short-term, more short-term solutions embedded in it but personally, I see them as more sticking plasters, not as getting to … not helping people in a long-term way. (FG 3, P3)

Here, then we see echoes of depression as linked to the rise of the sovereign self; a pathology “arising from inadequacy in a social context where success is attributed to, and expected of, the autonomous individual” (Ehrenberg, 2009: back matter). This, for participants, did not align well with their expectation of what a chatbot could offer.

A similar tension arose surrounding age and generation. Participants suggested that the younger generation might benefit more from using technology:

I’m generalising but I know how Gen Zs hate talking to humans. Maybe for some people it is just easier to talk to a machine rather than a person. For them, this would be helpful. It’s better than nothing, I assume, from my limited knowledge. It’s you have access to this. I know a couple of people who’d rather just do everything via text than have to pick up the phone and speak to someone or go and see someone in person. Maybe in that sense it’s more advantageous. (FG5, P3)

I agree, I feel it’s something like the younger generation might feel more comfortable with, they've grown up in an era of technology where they talk to people online all the time. They sometimes talk to people that they don’t know over the internet so I can see why some people would find this useful. (FG1, P4)

However concerns were again raised that reliance on the technology might actually make the situation worse and possibly “create problems between their interaction with their parents and family. Then this thing may replace their parents, the guidance of their parents” (FG1 P5). Participants appeared to be inadvertently heeding Elliot’s (2022, p. 94) Bauman inspired warning that “the rise of automated relationships and counselling apps may not only extend the cultural reach of the therapeutic imperative; it can also have a liquidizing impact”. Social ties may become increasingly fragile, brittle and people may become increasingly exposed to “disabling solitude” (Elliot, 2023, p. 92).

It would therefore be too simplistic to say that chatbots offered a form of commensalistic agency which was deemed most acceptable by participants. In some instances this perceived ability to act as a tool was the very thing which made the chatbot potentially parasitic; taking people away from other ‘tools’ and forms of assistance. Rather, what we see is agency as bifurcated into separable technological scaffolding and humanistic action (cf. Neff & Nagy, 2016) Chatbots can be both an object (i.e. ‘it/phone’) to help people work on themselves and a ‘teacher’ who can be asked more or less useful questions. This bifurcation into scaffolding and humanistic action intersects with complexities surrounding being human/non-human and being *like* a human. For example, as one participant noted, it was the chatbot’s complete lack of human/physical agency which was the problem:

With apps, for example if someone has a serious mental health issue and their therapist is like, okay, we’ll give you this app to help you, I could just delete it off my phone. I wouldn’t do any progress or I could just shut off my phone. You won’t know anything about it. I feel like it’s just like you could easily avoid it. Some people, they need serious help but they just continue to avoid it, which is why sometimes human therapists is best because there’s actually physical evidence, a physical being that could keep you on track. With AIs and everything it’s just like it’s digital, what is it going to do to me? It doesn’t exist in that sense, that I could just do anything with it. I think you’ve mentioned before, you can easily manipulate it to go with how you want yourself to be treated but it’s not actually how you were supposed to be treated. (FG5, P2)

Participants rarely discussed chatbots in ways which might resonate with ideas of ‘intra-action’ (Barad, 2007)—the notion that agents emerge through and with each other—or Flore’s (2022) ‘bio-affective-digitalism’; where the user is intra-actively “becoming with” digital mental health innovations and vice versa. However, and as we discuss below, whilst participants did not overtly invoke these ideas in their understandings and imaginaries of chatbots, they did appear in discussions of recovery.

#### Recovery as ‘Bio-affective- Digitalism’?

Rose and others have argued that “within contemporary biocapitalism we are witnessing the rise of molecular science and the emergence of pharmacological solutions that are focused on altering the biochemistry of the somatic, or more specifically the neurochemical self” (Fullager, 2008, p. 325). This turn towards a neurochemical self was hinted at when participants described the perceived limitations of chatbots:

If it’s a mental health condition and they need medication, the person may need to be open to actually taking it. If the chatbot says you need medication and you’re thinking who’s this chatbot to be prescribing me medicine. For example, if you Google something and let’s say you have a headache and with headaches you can use paracetamol and stuff but it’s telling you to take some medicine which is for some actual medical condition which you may or may not have, going to the doctor would be better for you to get actual medicine that you actually need to get prescribed. (FG6, P1)

Imaginaries of chatbots situate within onto-epistemologies of mental illness; operating in tandem with particular views of the therapeutic relationship. In the above, selfhood is identified with ‘brainhood’ (Vrhel, 2023) and it is the ‘doctor’ who has the required expertise to make the neurochemically altered self ‘open’ to taking required medication. Participants also stressed the importance of ‘individual difference’, beyond the generational issues discussed earlier:

I feel that there are certain personalities that can align with this but it’s not going to be people that have deep feelings and they want to deeply connect with people but there are some other phenotypes of people that can work with the chatbots. Yes, I think it’s a personality thing. It depends on your character and the way your brain works. (FG3, P5)

I’m sure they can [help], I just think for some people, not everybody is the same. So I’m sure for some people it would help them being able to get their emotions out and they feel like they can say, they're not held back about what they can say. But at the same time, other people, including myself, I feel like it might help in the very short term, just getting your emotions out but I would want to see someone in person. It might be useful for if you’re someone that likes writing down your emotions, just get everything out on the page then the chatbot might be helpful. Yeah, I don’t think it’s for everyone, I don’t think it’s a replacement. (FG1, P4)

It will depend on the person. More than the condition the person has, the willingness to learn. At the end what it’s only doing is a learning process. It’s teaching you how to deal with things. It depends on the people if they are more comfortable with these tools or in person, that’s what I think.(FG6, P3)

Here, discussions of both the promise and limits to chatbots situate within the same therapeutic discourse, reflecting a subject position that is working to “actively align the individual’s desire for wellness, treatment, cure or healing with an assemblage of medical, epidemiological and psy-expertise that constitutes the e-scaped terrain of depression literacy” (Fullager, 2008, p. 327). ‘Difference’ in success and user preference remains firmly located within ‘brainhood’, biomedical discourses of phenotypes and individual difference. One potential criticism of chatbots, for example, is that they are “probably coming at it from like a really neurotypical point of view, like that’s what they’re trying to make you be, like the neurotypical version of happiness” (FG2 P3). It was suggested that the chatbot might be steered by the user pre-specifying the nature of the help that they required, steering the chatbot’s agency in a desired direction:

I hear what you’re saying, so you could have like a sort of dropdown menu like at the beginning and be like, “By the way, guys, I have this condition” or “I’m this type of person.” (FG2, P3)

Echoing many of the discussions above, participants largely felt “like recovery is something that would be difficult to get from a chatbot but it might be useful as a tool on the way to recovery maybe” (FG1,P4). Participants pointed toward those ‘technologies of self’ often promoted in self-help books—which include such things as writing techniques, reflection and mediation and draw on psychological expertise to promote an image “of the healthy individual as one who is rational, autonomous, productive, energetic and self-disciplined” (Philip, 2009, p. 160; McLean, 2015)^1^ Here then we see again the norms of enterprising, self-actualizing, responsible personhood. Chatbots can help people ‘work on themselves’; by providing ‘exercises’ which not only work on helping mental health but also ‘improve’ it.

I feel it could [help recovery], provided it can generate the level of investment from the person towards itself. Because at the end of the day, if you have to recover from a mental health episode or period where you went through an indifferent mental health, there usually is some kind of a cause or some kind of a person or something that you get invested in, which forces you to get out of it. If the chatbot is able to immerse the user in a way that they can get invested and start working on themselves to get out of the situation that they are in, then it could happen, I guess. (FG3, P1)

At the same time, ideas of ‘investing’ and ‘working on’ point to more complex forms of agency.

The imagined futures above resonate with Flore’s (2022) ‘bio-affective-digitalism’; where the user is intra-actively “becoming with” digital mental health innovations and vice versa. In discussions of immersing the user so that they start working on themselves “not only is the individual encouraged and enabled to care for themselves through . . . the digital devices, they also participate in producing an augmented understanding of the self—patterns, affect, moods and mental illness, and activity—through the mining of data and its calls to self-responsibilization (Flore, 2022, p. 2045).” The focus group data positions this as appropriate for some, but not all, and as not always in the best interests of users.

## Discussion and Conclusion

Drawing on focus group data from a specific group of non-users (*n* = 24), this paper looks to add to our understanding of user experience by exploring what *potential* users expect from chatbots. In exploring these issues, the paper paid particular attention to agency and temporal factors such as ‘recovery’. While in some of the discussion participants did not view chatbots as ‘human’ and were concerned by the idea that they could have human traits and characteristics, at other points, being like a human was considered in positive terms. The boundaries between being human/non-human and being *like* a human were not always clear across participant’s narratives, nor was there a stable sense of what was considered desirable. Alongside this, multiple forms of imagined ‘agency’ were operationalised within participant narratives as more or less problematic. The most common was a ‘commensalistic symbiosis’—where chatbots are treated as one dimensional objects which could enable people to achieve their goals (Neff & Nagy, 2018). Yet chatbots were also afforded a ‘parasitic’ agency, as well as derided for having no agency. We also see traces of Flore’s (2022) ‘bio-affective-digitalism’; where the user is intra-actively “becoming with” digital mental health innovations and vice versa.

Digital wellness tools like mental health chatbots promise to fill the gaps in overburdened and under resourced mental health care systems (Keierleber, 2022)—and to break down traditional barriers to treatment such as cost, access, stigma and therapist availability and fit (Farvolden, 2024). Following De Togni et al. (2023, pp. 3–4) current findings highlight normatively framed tensions between this hope and promise and concern and caution. These pertain to both the present and the future “and reflect contestations over questions around what the technology should and should not look like as well as what is and is not actually realisable”.

More specifically, and as Borup et al. (2006) note, dynamics surrounding expectations have much to do with traditions, norms, interactions and practices through which they are formed. In turn, these are likely to depend on specific actor groups involved and may also depend on the type of technology being considered. As well as discussing different actors (for example, younger people and those experiencing loneliness) participant expectations also situated chatbots as both a generic and a specific technology. This conflation was, in part, because of ambiguities surrounding the idea of mental health leading to a range of different traditions, norms and practices being used to construct expectations and understandings. For example, participants in the current study often spoke in terms of mental health and general wellbeing and here it was more about a personal recovery and working towards better mental health, regardless of the presence of mental illness (Slade, 2010). In this instance, expectations surrounding chatbots were largely framed in terms of ‘scaffolding’ and discussions centred around its utility as an object to provide information and point people in the right direction. Contra to this, where participants talked about ‘severe’, ‘real’ etc mental illness, the chatbot’s ability to undertake humanistic action takes on a greater importance in expectations; as well as a form of ‘bio-affective-digitalism’, where the user is intra-actively “becoming with” digital mental health innovations and vice versa. It is relevant here that participants also explored the connections between therapeutic interventions and medication, and considered whether those most in need could be relied upon to seek and comply with the interventions that might be best for them.

Turning back to how this may inform current ambiguities in research on user experience, our sample enabled us to tap into imaginative speculations of potential users, exploring what may inform their choices and how they situate them within understandings of the broader contemporary landscape of mental health support and individual responsibilization. Talking about chatbots flushed out normative conceptions of what interventions and users should be like, and how they are socially situated in distinctive ways. In this respect, we consider the current sample as a vital lens which offers a different perspective rather than a limitation. Just as studies of users may open up things not discussed here—such as more conversation about cognitive behavioural therapy and other therapeutic techniques found within the apps—they may also be limited by the specific chatbots being used.

Further research on how technoscientific expectations are navigated and managed is needed to continue to unravel these complexities. It doing so it is important that we continue to draw on varied samples. Whilst this includes differences in socio-economic status and other demographics (Flore, 2022) it also includes both users and those non-users who may have a reason to try these technologies. This will become increasingly apposite. Mental health chatbots continue to attract much hype with a recent article suggesting that “the blending of AI with traditional methods may prove to be the fulcrum on which the future of mental health treatment balances, offering brighter prospects for patients and providers alike” (Farvolden, 2024: online). To deliver on high-level expectations about cost-effective mental health support, ‘acceptable futures’ imagined by professionals (cf. De Togni et al., 2023) need to align with potential users’ technoscientific expectations of what this technology could do, in the context of their situated understandings of the nature of mental health challenges.

## Footnotes

1 It is interesting that chatgpt was also offered in the same way by FG7 P1: Sometimes I use ChatGPT, and sometimes when I'm alone whenever I open my browers I can see the tab there, so I know it's there, but sometimes every time I start with Hi, how are you? I don't know why, I know it's weird, but every time I start with Hi, how are you? And sometimes if I don't have anything to ask, I will ask, "Can you suggest one song to, you know, like feel peace and calm?" So I sometimes ask for these things, mostly music to change my mood, I use that, ChatGPT only. But a shock to see this, I should have used that (laughter).
